# Analysis of Minimum Quantity Lubrication (MQL) for Different Coating Tools during Turning of TC11 Titanium Alloy

**DOI:** 10.3390/ma9100804

**Published:** 2016-09-28

**Authors:** Sheng Qin, Zhongquan Li, Guoqiang Guo, Qinglong An, Ming Chen, Weiwei Ming

**Affiliations:** 1 Department of Mechanical Engineering, Shanghai Jiao Tong University, Shanghai 200240, China; qinsheng@sjtu.edu.cn (S.Q.); qlan@sjtu.edu.cn (Q.A.); mingseas@sjtu.edu.cn (W.M.); 2 Shanghai Aerospace Precision Machinery Research Institute, Shanghai 200240, China; lizhongquan401@163.com (Z.L.); guoguo_0@163.com (G.G.)

**Keywords:** TC11 aluminum alloy, turning, minimum quantity lubrication (MQL), tool coating, tool wear

## Abstract

The tool coating and cooling strategy are two key factors when machining difficult-to-cut materials such as titanium alloy. In this paper, diamond coating was deposited on a commercial carbide insert as an attempt to increase the machinability of TC11 alloy during the turning process. An uncoated carbide insert and a commercial Al_2_O_3_/TiAlN-coated tool were also tested as a comparison. Furthermore, MQL was applied to improve the cutting condition. Cutting performances were analyzed by cutting force, cutting temperate and surface roughness measurements. Tool wears and tool lives were evaluated to find a good matchup between the tool coating and cooling strategy. According to the results, using MQL can slightly reduce the cutting force. By applying MQL, cutting temperatures and tool wears were reduced by a great amount. Besides, MQL can affect the tool wear mechanism and tool failure modes. The tool life of an Al_2_O_3_/TiAlN-coated tool can be prolonged by 88.4% under the MQL condition. Diamond-coated tools can obtain a good surface finish when cutting parameters and lubrication strategies are properly chosen.

## 1. Introduction

TC11 (Ti-6.5Al-3.5Mo-1.5Zr-0.3Si) is a kind of α + β two-phase titanium alloy mostly used in turbine blades for its high temperature resistance and creep strength, which also leads to its poor machinability. Like most other titanium alloys, the main issues of concern when machining TC11 include the extremely high temperature at the tool-chip interface, severe adhesion, premature failure of the cutting and poor surface finish [1]^.^

A tool material with high hardness values, low friction coefficients and superior thermal characteristics is an essential key factor when machining titanium alloy. Researchers have been devoted to finding more suitable coating materials for cutting tools. Carbide tools are still mostly used in the industry when machining titanium alloy. Liang et al. [2] investigated the possible beneficial effect of Ni_3_Al binder on WC-based cemented carbides by using tools prepared with the spark plasma sintering technique. By analyzing tool wear at different speeds, WC-10Ni_3_Al was proved to have a better performance. Nanocomposite coatings have been investigated and used widely in recent years for their extremely high hardness and heat resistance. Rodríguez-Barrero et al. [3] investigated the performances of several nanostructured coatings for drilling under high temperatures, including AlCrSiN, μAlTiN, TiAlCrN, AlTiCrN, AlCrN, AlTiSiN, and TiAlSiN. The results of flank wear, adhesion, chip evacuation and force show that TiAlSiN, AlTiSiN, and μAlTiN have better performances because of greater hardness, oxidize at higher temperatures and have low chemical affinity. It should be noted that all the coatings were applied by a post-process named “drag grinding” before the test, which leads to an increase of tool life by up to 30%. Liu et al. [4] studied the wear performance of (nc-AlTiN)/(a-Si_3_N_4_) coating and (nc-AlCrN)/(a-Si_3_N_4_) coating turning Ti6Al4V and obtained a good surface finish by applying the minimum quantity lubrication (MQL). Normally, diamond tools are not suggested when machining titanium alloy due to its high chemical activity and graphitization. However, researchers have applied diamond-coated tools for certain circumstances. Minton et al. [5] performed new-dry cutting with a specially designed internally cooled diamond-coated tool, resulted in a long tool life while maintaining high standards of the surface finish.

The cooling strategy plays a vital role when machining difficult-to-cut material. Dry and minimum quantity lubrication (MQL) have been proposed and practiced more often when sustainable machining is concerned. Great efforts have been made to study the MQL effect in different machining processes [6,7,8,9,10,11]. The materials of the mist, cooling devices and parameters play the most important roles. Kamata et al. [10] performed a finish turning process on Inconel 718 with different coating tools and attained a better tool life and surface finish than that in the wet and dry machining. Deiab et al. [11] used vegetable oils in MQL and compared the tool wear, surface roughness and energy consumption of different cooling strategies during the turning of Ti6Al4V and proved MQL to be an overall sustainable alternative. Dry cutting and MQL are limited to workpiece materials and to reduced cutting parameters [12]. When machining high-temperature alloys, other than cooling and lubrication effects, chip breaking is also of great importance. High-pressure cooling is an alternative for good chip breaking, especially with carbide inserts. Busch et al. [13] applied high-pressure cooling with ceramic inserts when machining Inconel 718. The tool life was slightly poorer and chip breaking was improved comparing to conventional cooling, which shows that high-pressure cooling can be used in the machining of heat-resistant alloys with ceramic tools. Liquid carbon dioxide (CO_2_) or nitrogen (LN_2_) are often used in cryogenic cooling as an alternative to high-pressure cooling [12]. Bruschi et al. [14] investigated the influence of the machining parameters and cooling strategies on the reciprocating sliding wear behavior of the Ti6Al4V titanium alloy. The results show that by using cryogenic cooling, lower friction and less abrasive wear were achieved. Pereira et al. [15] proposed an alternative named ‘CryoMQL’ to minimize the use of coolant by applying both cryogenic gases and MQL turning AISI 304 stainless steel. Experimental results show that tool life is exceeded by 30% compared with MQL and doubled with dry machining. Besides, the cutting forces and surface integrity are not compromised. CryoMQL provides a solution for technical and environmental issues during wet and dry machining of difficult-to-cut materials and provides the possibility of increasing the tool life and cutting efficiency at the same time.

Therefore, tool coating, tool selection and cooling strategy are two key factors when machining titanium alloy. Besides, the match-up between the tool coating and lubrication strategy is vital and was not often considered in combination in previous work. So it is important to perform a study taking into account different lubrication strategies in order to obtain the best choice of tools when the tool life or integrity of the component is considered. This paper evaluated the cutting performance of carbide, Al_2_O_3_/TiAiN-coated and diamond-coated tools in the turning of TC11 under dry and MQL conditions, with cutting force and temperature, tool wear and tool life, and surface integrity in terms of roughness being compared as tool selection criteria.

## 2. Experimental Details

### 2.1. Test Material

The workpiece used in the turning experiments was a TC11 (Ti-6.5Al-3.5Mo-1.5Zr-0.3Si) bar with the size of Ф120 mm × 100 mm. The material mainly consists of alpha phase, with the grain size of 5 μm, ratio of 0.7. The rest is the beta phase. Both phases consist as equilibrium at room temperature. The chemical composition (wt %) and mechanical properties of TC11 are presented in Table 1 and Table 2.

### 2.2. Cutting Tools

An uncoated carbide insert (CNMG120408-SMR H13A, Sandvik Coromant, Trosa Municipality, Sweden), a physical vapor deposition (PVD) (Al_2_O_3_/TiAlN)-coated inset (CNMG120408-SMR 1115, Sandvik Coromant, Trosa Municipality, Sweden), and a chemical vapor deposition (CVD) diamond-coated insert were adopted to make comparisons during the experiments. The diamond-coated insert was obtained by coating diamond on the commercial carbide insert (CNMG120408-SMR H13A).

The diamond films were deposited by using a bias-enhanced hot filament chemical vapor deposition (HFCVD) apparatus. Flat square shaped WC-6%Co inserts were used as substrates. Preceding the deposition, the WC-Co substrates were dipped in the Murakami’s reagent (10 g K_3_[Fe(CN)]_6_ + 10 g KOH + 100 mL H_2_O) in an ultrasonic vessel for 30 min to roughen the substrate surface. Then the substrates were etched with Caro’s acid (30 mL HCl:70 mL H_2_O_2_) for 1 min to reduce the surface cobalt concentration which will induce interfacial graphitization. After etching, seeding is accomplished by using an ultrasonic treatment of the tool in a diamond powder and acetone solution. A nano-diamond (4–10 nm) powder synthesized using the detonation technique is used to make the solution, as it is known to be successful at creating thin, conformal diamond coatings. Two pair-twisted tantalum wires were used as hot filaments and fixed in parallel with a distance of 40 mm. The tool is mounted in a supporting stick which is in the middle of the filaments and the tool nose is positioned 2 mm below the filament. During the deposition, the temperatures of hot filaments and the substrate surface were 2200 °C and 800 °C, respectively.

**Figure 1:**
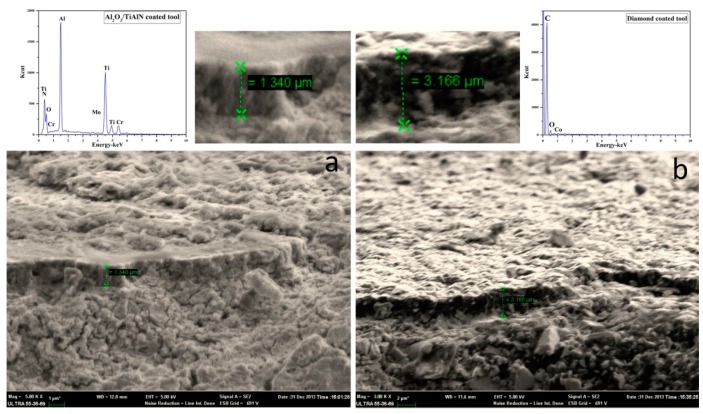
SEM micrographs and EDS analysis of the coating surfaces ((**a**) PVD (Al_2_O_3_/TiAlN)-coated insert; (**b**) CVD diamond-coated insert).

For observation of the coating, the coated tools were cross-sectioned perpendicular to the rake face. The SEM micrographs and EDS results of the tool surface illustrated in Figure 1. The thickness of the PVD-coated tool and CVD-coated tool was 1.340 μm and 3.166 μm, respectively.

### 2.3. Global Scheme

**Figure 2:**
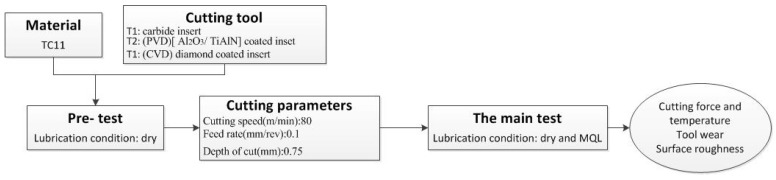
Global scheme of the experiment.

The global scheme of the paper is shown in Figure 2. Fernandez-Valdivielso et al. [16] proposed an indirect approach based on waterfall filtering to find the influence of tool features on integrity of components when machining Inconel 718. Similar to the raw test of their work, a pre-test using Taguchi method was conducted in order to select proper tools and cutting parameters for production, in consideration of tool life and productivity, which is also the ones used in the main test. Details of the pre-test would not be discussed in this paper.

The cutting parameters used in the main test are listed in Table 3. The main purpose of the test in to find the match-up between tool coating and lubrication strategy. Dry cutting and MQL were conducted in the main test. Cutting force and temperature were tested, which gave the base for better understanding the tool wear mechanism. Tool life and surface integrity in terms of surface roughness are two main targets for both process and experimental consideration.

### 2.4. Experimental Specifications

**Figure 3:**
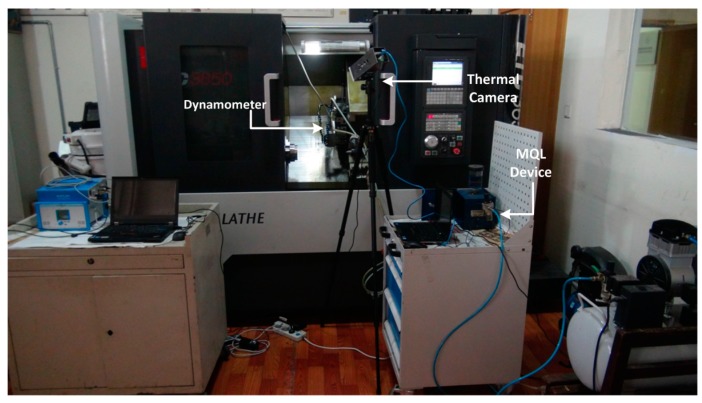
Experimental set-up for turning TC11.

The experiments under dry and MQL condition were performed using an ETC3650U (Shenyang No. 1 Machine Tool Sales Co., Ltd., Shenyang, China) turning center. Seen from Figure 3, a Kistler 9129AA (Kistler, Winterthur, Switzerland) dynamometer was mounted under the workpiece to measure the cutting force. To investigate the coolant effect of MQL, a FlirA615 (Behringer Eurolive, New York, NY, USA) thermal camera with resolution of 640 × 480 pixels and thermal sensitivity of 50 mK was used. The other details of the experiment are listed in Table 3.

## 3. Result and Discussion

### 3.1. Cutting Force and Temperature

**Figure 4:**
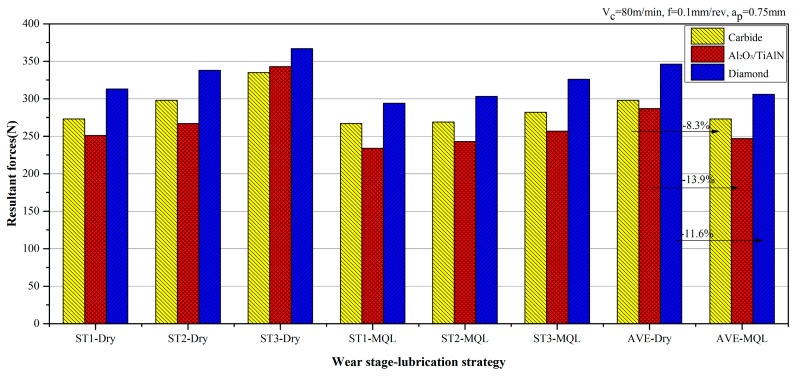
Cutting force comparison under dry and MQL conditions.

Cutting forces were measured during the experiments with the cutting parameters given in Table 3. The resultant forces (*F_R_*) during three tool wear stages (ST1: initial wear stage, ST2: steady wear stage, ST3: intensive wear stage) and the average one of the whole cutting time duration (AVE) under dry and MQL conditions are compared in Figure 4. For either dry or MQL conditions, the lowest forces were obtained with the Al_2_O_3_/TiAlN-coated tools and the highest with the diamond-coated tools. This might be the result of both the coating friction reduction and contact radius incensement. Comparing to the carbide tool, the Al_2_O_3_/TiAlN coating with a thickness of 1.340 μm, obtained by the PVD process, has a lower friction coefficient and similar tool contact radius. The diamond tool was coated beyond the carbide tool by the CVD process, with a thickness of 3.160 μm, which increases the contact radius of the tool by a great amount and led to much greater cutting forces. It also can be seen from Figure 3 that for the same cutting tool, when MQL was applied, the resultant forces were reduced for a certain amount, ranging from 8.3% to 13.9%.

**Figure 5:**
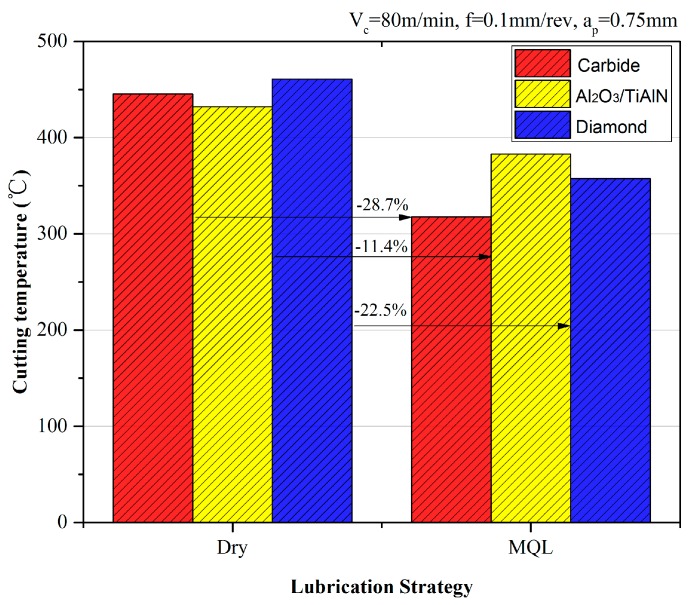
Cutting temperature comparison under dry and MQL conditions.

Figure 5 shows the effect of MQL on the cutting temperature and different tools. It can be seen that for the same lubrication strategy, the lowest temperature was obtained with the Al_2_O_3_/TiAlN-coated tool and the highest with the diamond-coated tool. Besides, by using MQL, the cutting temperatures were reduced to a greater extent compared to the cutting forces, ranging from 22.5% to 28.7%.

### 3.2. Tool Wear Mechanism

**Figure 6:**
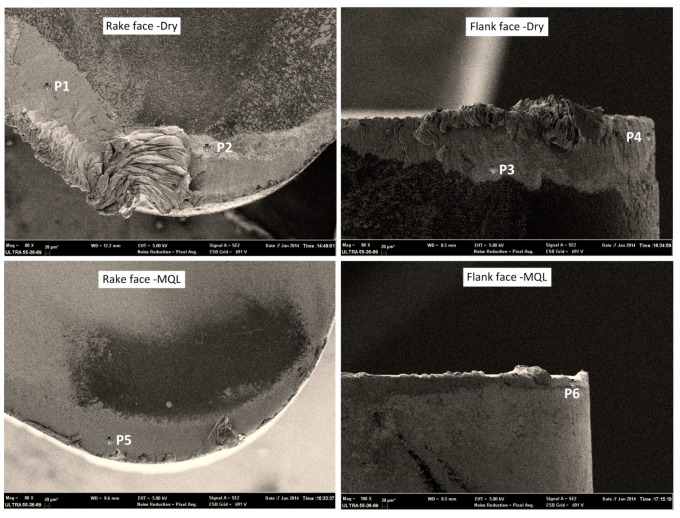
SEM images of carbide insert under dry and MQL conditions.

Figure 6 and Table 4 show the SEM micrographs and EDS analysis of the wear track at the rake and flank face of the carbide tools under dry and MQL conditions. For both conditions, the adhering materials smear on the rake face, which is proved to be TC11 transferred from the chip material. The amount of the deposited material on the rake face is greater than the flank face, which is caused by the higher temperature of the rake face. With the relative movement of the chips, workpiece material and tool, grains of the tool material adhered and were taken from the tool, causing the adhesion wear. By comparing the rake and flank faces of the tool under two lubrication conditions, using MQL led to a decrease in the amount of the layer adhered to the tool. Moreover, the presence of the C element in these adherent layers (seen in Table 4) is evidence of the diffusion of carbon from the tool material. Under the dry condition, the deposited layer can be found and the fresh surface is presented, which reveals that the tool substrate strength decreased and thereby the tool material was torn and taken away. On the other hand, the main wear pattern on the rake face of the carbide tool under the MQL condition is adhesive wear, less severe than that of the flank face, and abrasive wear, which can be seen by the clear abrasive marks. This phenomenon is also evidence that MQL can reduce the adhesive wear.

**Figure 7:**
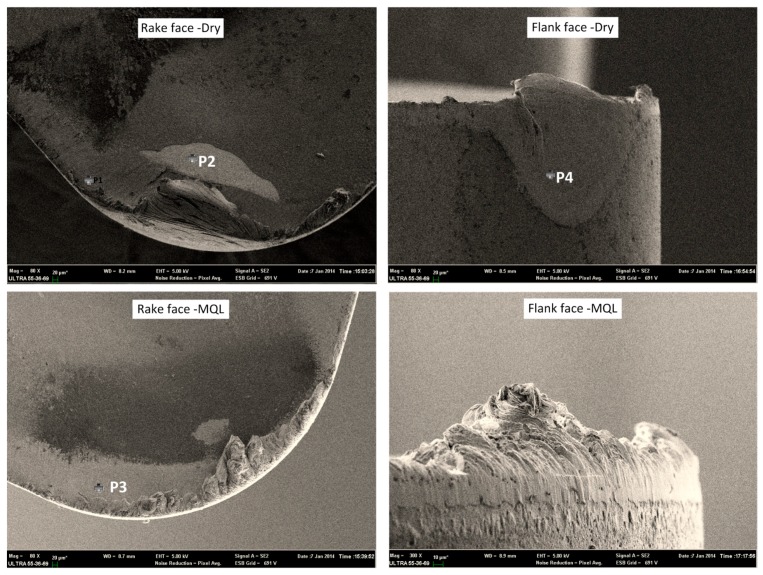
SEM and EDS results of Al_2_O_3_/TiAlN-coated insert under dry and MQL conditions.

The SEM images of a worn Al_2_O_3_/TiAlN-coated tool in combination with EDS are shown in Figure 7 and Table 5. Under the dry condition, the tool failed in the form of the coating peeling off and chip breaking, which is probably due to the force and thermal impact during machining. The adhesive layer was peeled off the tool. The EDS results show evidence of adhesion and diffusion. Compared to the carbide tool, the presence of the O element in a higher amount indicates more severe oxidative wear, which was probably caused by the higher temperature.

The main wear mechanism is still the adhesive wear on the rake face, and the adhesive and abrasive wear on the flank face. The deposited layer can be clearly seen on the rake and flank faces and EDS results show similar patterns. By applying MQL to the turning operation, the temperature was reduced and premature tool failure was avoided.

**Figure 8:**
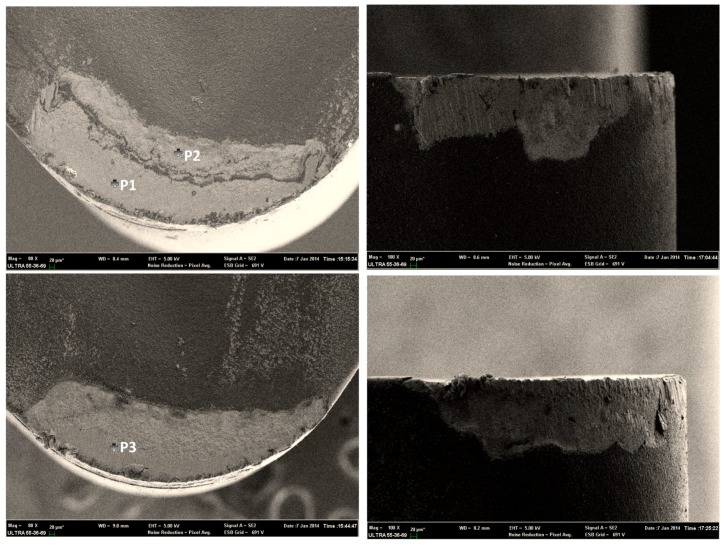
SEM and EDS results of diamond-coated insert under dry and MQL conditions.

Figure 8 and Table 6 show the SEM and EDS results of worn diamond-coated tools. Under both dry and MQL conditions, the coatings were peeled off. Due to the high temperature when machining TC11, oxidization and graphitization of the coating material led to coating failure and a substrate whose strength was weakened during the coating process participated in the machining, which led to more severe tool wear. However, the adhesion of the rake face was proved during the lower friction, but it is still the main wear pattern. The cooling effect of the MQL under the condition of the trial turned out to be ineffective enough to avoid the peeling of the coating.

It can be concluded that the main wear mechanism of the tools when turning TC11 is adhesive and diffusive wear on the rake face, and adhesive and abrasive on the flank face. The effect of MQL is mainly on the insensitivity of the wear, which could result in changing the tool wear rate and failure mode.

### 3.3. Tool Life

**Figure 9:**
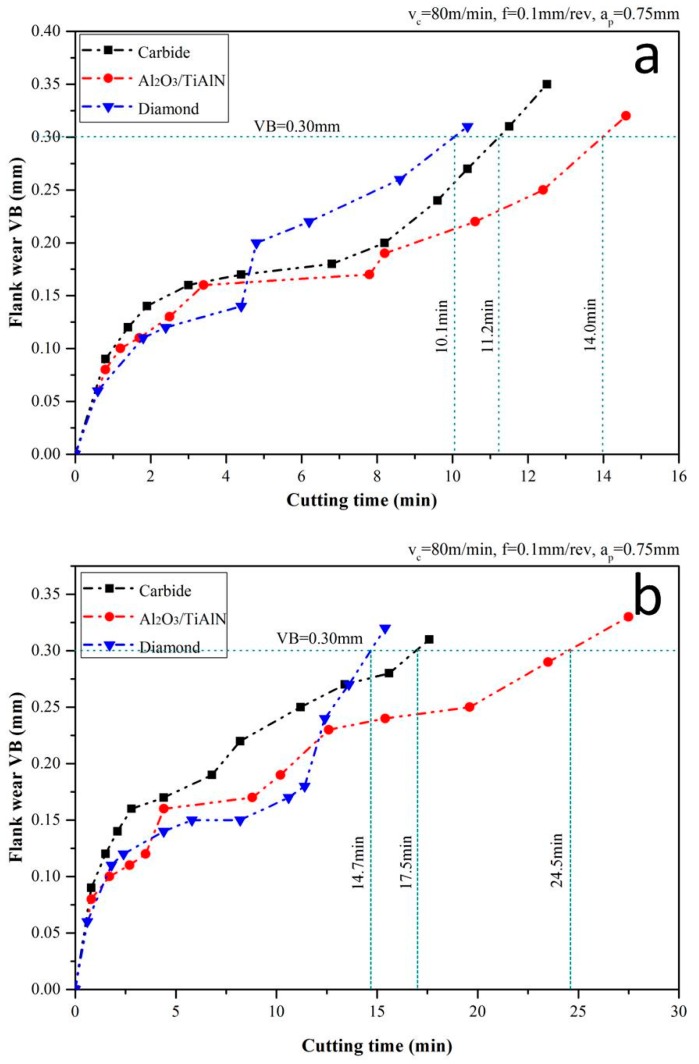
Tool wear under dry (**a**) and MQL (**b**) condition.

During the experiments, the average flank wear VB versus the cutting time for each tool at a cutting speed of 80 m/min under dry and the MQL condition was measured and is depicted in Figure 9. Under the dry condition, the diamond tool underwent the longest initial wear stage and a small period of the steady stage. At the time of 4 min, the diamond coating on the cutting edge desquamated and the substrate with lower strength compared to the carbide tool (the result of coating pre-processing) participated in the cutting process. As a result, the tool wear accelerated and the shortest wear period among the three tools was obtained. This is understandable because the highest cutting force and temperature of the tool (shown in Section 3.1) result in oxidization and graphitization of the coating material. It also can be seen from Figure 8 that at the time of 8 min, the tool wear of Al_2_O_3_/TiAlN-coated tool suddenly got into the intensive wear stage, which is the result of the coating peeling off and cutting edge breakage as mentioned in Section 3.2. However, the Al_2_O_3_/TiAlN-coated tool still obtained the longest wear period, which indicates that the coating is suitable for the TC11 turning process under the cutting condition of this paper. From the result in Section 3.2, we can see that, under the MQL condition, the diamond-coated tool still failed because of the coating peeling. However, the steady wear period of the diamond tool was prolonged to the time about 12 min, before which the wear of the tool is quite low. The effect of MQL on the Al_2_O_3_/TiAlN-coated tool was reflected in the lower wear of the initial stage and the longer duration of the steady wear stage. Besides, from both the curve and result in Section 3.2, we can infer that MQL has the effect of avoiding edge breakage and coating peeling through decreasing the force impact and temperature.

**Figure 10:**
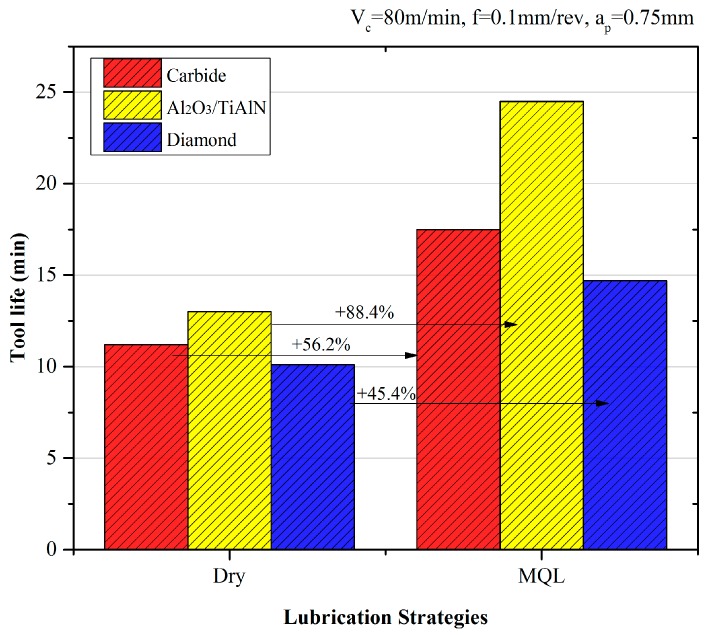
Tool life comparison of tools under dry and MQL conditions.

The tool life comparison of different coating tools under two lubrication conditions are exhibited in Figure 10. The diamond tool obtained a comparable tool life under the MQL condition. It is also noted that the MQL exceed the tool life by 88.4% for the Al_2_O_3_/TiAlN-coated tool, which allows us to infer that the MQL condition has a significant effect on improving the tool life when the coating is chosen properly.

**Figure 11:**
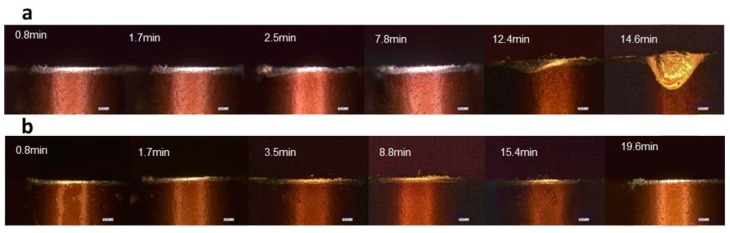
Optical images of tool wear and failure of Al_2_O_3_/TiAlN-coated insert ((**a**) under dry cutting; (**b**) under MQL).

Figure 11 shows the optical images of tool wear and failure of the Al_2_O_3_/TiAlN-coated insert. During the steady wear stage, adhesive material was found on the cutting edge under the dry condition from t = 0.8 min to the time of tool failure. On the other hand, the tool edge was comparably clean under the MQL condition. Thus, the difference of tool wear during the steady stage is quite obvious. When the cutting time reaches 12.4 min, tool failure in terms of breaking can be seen from Figure 11a under the dry condition, while during the same time period, the tool under MQL was still undergoing steady wear. Similar to the result given by SEM and EDS tests, by using MQL, adhesive wear was reduced and premature tool failure such as chip breaking could be avoided.

**Figure 12:**
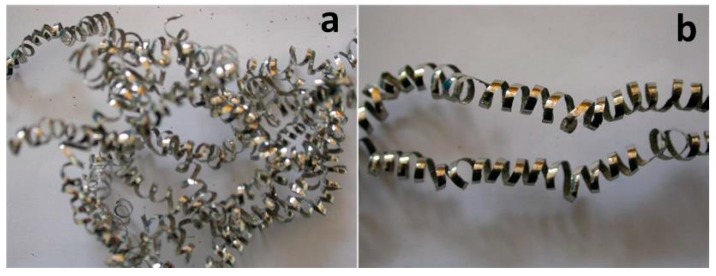
Optical images of tool wear and failure of Al_2_O_3_/TiAlN-coated insert ((**a**) under dry cutting; (**b**) under MQL; cutting time t = 12 min).

Furthermore, Figure 12 shows the optical images of chips removed by the Al_2_O_3_/TiAlN-coated tool at cutting time at about 12 min under dry and MQL conditions. It can be observed that the chip produced under the dry condition is tangled, while the chip produced under the MQL condition is regular. The adhesive material peeling from the insert caused uneven voids on the rake face and uniform build-ups on the cutting edge, which produced an unsmooth back face and an uneven curvature of the chip. This result further proves that by using MQL, the wear condition of the cutting tool can be improved.

### 3.4. Surface Roughness

**Figure 13:**
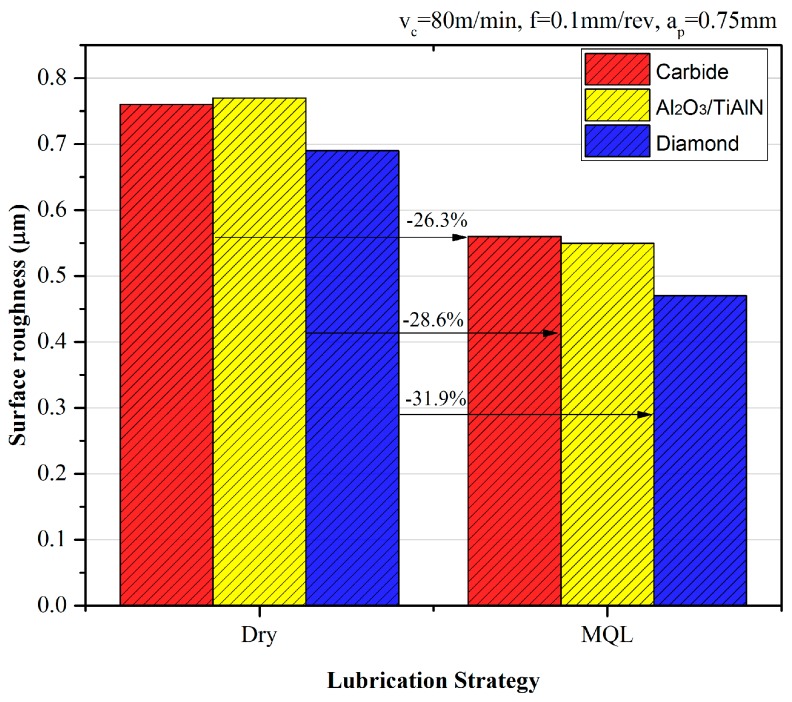
Surface roughness comparison for different tools under dry and MQL conditions.

Machined surfaces were measured to analyze the integrity in relation to the roughness (*R_a_*). Tests were carried out three times for each surface and the mean values are shown in Figure 13. Under dry and MQL conditions, the diamond-coated tool provided the best surface finish even though the tool wear was intensive. This is probably due to the smooth and low friction surface of the diamond coating. Besides, the contact radius of the tool is the greatest, which is negatively correlated to surface roughness. Further, consider that the diamond-coated tool can obtain a satisfactory tool life when MQL is applied (seen in Section 3.3). When the surface roughness is mainly considered, the diamond-coated tool can be a choice depending on the condition that the cutting parameters and cooling strategy are chosen properly. It also can be seen in Figure 10 that by using MQL, the surface roughness can be reduced by about 30%. The diamond-coated tool obtained the highest reduction of 31.9% and the PVD-coated tool the second highest reduction, which is probably due to the elongation of the steady wear state which results in better tool wear.

## 4. Conclusions

TC11 titanium alloy has been machined under dry and MQL conditions using a carbide tool, an Al_2_O_3_/TiAlN-coated tool and a diamond-coated tool, aimed at investigating the influence of cooling strategies on the performance of different coating tools. Cutting force, cutting temperature, wear behavior and surface roughness were tested and analyzed. The following conclusions can be drawn:

Under the same cooling condition, the lowest forces were obtained with the Al_2_O_3_/TiAlN-coated tools and the highest with the diamond-coated tools, which might be explained by the differences in the friction coefficient of the coating material and the contact radius of the inserts. When the cutting forces are mainly concerned, PVD-coated tools with a low friction coefficient and small contact radius are recommended. The cutting forces under MQL are slightly smaller than those under dry cutting. The decrease is about 10%. Compared to the forces, by using MQL, the cutting temperatures were reduced to greater extents.

The main wear mechanism of the tools when turning TC11 is adhesive and diffusive wear on the rake face, and adhesive and abrasive wear on the flank face. By using MQL, the amount of TC11 material deposited on the tool faces can be significantly reduced. As mentioned above, MQL has a great influence on the cutting temperature at certain cutting parameters, and the occurrences of tool failure such as chip breaking and coating peeling off can be reduced by using MQL. The Al_2_O_3_/TiAlN-coated tool was proved to be a better choice when tool life is concerned. Under MQL, the tool life can be prolonged up to 88.4%.

The diamond-coated tool can obtain a good surface finish under the MQL condition. The main effect of MQL is to reduce the cutting temperature. Oxidization and graphitization of the diamond coating material are restrained to a small amount. As a result, the tool life can be prolonged to a satisfactory duration. It can be concluded that when the cutting parameters and cooling strategy are chosen properly, the diamond-coated tool can be used for the purpose of obtaining a good surface finish when turning TC11 titanium alloy.

## Figures and Tables

**Table 1:** Chemical composition of TC11.

Al	Mo	Mn	Si	Zr	Ti
5.8~7.0	2.8~3.8	0.8~2.0	0.2~0.35	0.15	Remainder

**Table 2:** Mechanical properties of TC11.

Tensile Strength (25 °C)	Tensile Strength (500 °C) [MPa]	Yield Strength (25 °C) [MPa]	Elongation (Over 2 Inches) [%]	Hardness	Elastic Moduler [GPa]
1030	685	1000	10	0.15	110

**Table 3:** Experimental specifications.

Machine Tool	ETC3650U
**Cutting Parameters**	Cutting speed (m/min): 80 Feed rate (mm/rev): 0.1 Depth of cut (mm): 0.75
**Cutting Tool**	T1. CNMG120408-SMR H13A; T2. CNMG120408-SMR 1115 T3. CNMG120408-SMR H13A (Coated with diamond)
**Dynamometer**	Kistler 9129AA
**Thermal Camera**	FlirA615

**Table 4:** EDS analysis of the wear region of the carbide insert (wt. %, check points refer to Figure 5).

Point	C	Al	Ti	Zr	W	O	S	V	Cr	Co	Mo
1	3.27	4.87	88.59	1.79	0.37	-	-	-	-	-	-
2	6.11	1.54	31.49	0.84	57.41		1.54	0.34	0.08	0.16	-
3	5.17	0.40	5.61	1.23	77.53	0.82	-	-	-	0.92	0.53
4	-	5.32	86.48	1.13	0.68	0.45	-	0.35	-	0.19	2.58
5	1.87	1.29	19.14	1.25	72.62	0.33	-	-	-	1.05	2.46
6	-	3.28	86.41	1.91	-	-	-	0.67	-	0.26	3.56

**Table 5:** EDS analysis of the wear region of Al_2_O_3_/TiAlN-coated insert (wt. %, check points refer to Figure 6).

Point	C	Al	Ti	Zr	W	O	S	V	Cr	Co	Mo
1	-	2.77	28.07	0.91	62.27	1.22	-	0.16	-	4.55	1.03
2	8.64	0.59	1.18	-	76.99	1.72	-		1.12	8.59	-
3	0.98	3.25	61.70	0.46	30.85	-	-	0.15	-	0.93	1.67
4	0.01	0.67	10.74	1.02	75.01	1.72	-	-	-	4.40	0.51

**Table 6:** EDS analysis of the wear region of the carbide insert (wt %, check points refer to Figure 6).

Point	C	Al	Ti	Zr	W	O	S	V	Cr	Co	Mo
1		5.22	84.69	2.49				0.42		0.29	6.14
2	5.22	0.84	10.66	0.32	79.57	2.07		0.20		0.46	0.65
3	5.11	1.02	18.80	0.85	67.75	0.23		0.12		0.30	1.08

## References

[1] Çalışkan H., Küçükköse M. (2015). The effect of aCN/TiAlN coating on tool wear, cutting force, surface finish and chip morphology in face milling of Ti6Al4V superalloy. Int. J. Refract. Met. Hard Mater..

[2] Liang L., Liu X., Li X., Li Y. (2015). Wear mechanisms of WC–10Ni3Al carbide tool in dry turning of Ti6Al4V. Int. J. Refract. Met. Hard Mater..

[3] Rodríguez-Barrero S., Fernández-Larrinoa J., Azkona I., de Lacalle L.N.L., Polvorosa R. (2016). Enhanced performance of nanostructured coatings for drilling by droplet elimination. Mater. Manuf. Proc..

[4] Liu Z., An Q., Xu J., Chen M., Han S. (2013). Wear performance of (nc-AlTiN)/(a-Si_3_N_4_) coating and (nc-AlCrN)/(a-Si_3_N_4_) coating in high-speed machining of titanium alloys under dry and minimum quantity lubrication (MQL) conditions. Wear.

[5] Minton T., Ghani S., Sammler F., Bateman R., Fürstmann P., Roeder M. (2013). Temperature of internally-cooled diamond-coated tools for dry-cutting titanium. Int. J. Mach. Tools Manuf..

[6] Le Coz G., Marinescu M., Devillez A., Dudzinski D., Velnom L. (2012). Measuring temperature of rotating cutting tools: Application to MQL drilling and dry milling of aerospace alloys. App. Therm. Eng..

[7] Sharma A.K., Tiwari A.K., Dixit A.R. (2016). Effects of minimum quantity lubrication (MQL) in machining processes using conventional and nanofluid based cutting fluids: A comprehensive review. J. Clean. Prod..

[8] Sun Y., Huang B., Puleo D.A., Jawahir I.S. (2015). Enhanced Machinability of Ti-5553 Alloy from cryogenic machining: Comparison with MQL and flood-cooled machining and modeling. Procedia CIRP.

[9] Uysal A., Demiren F., Altan E. (2015). Applying minimum quantity lubrication (MQL) method on milling of martensitic stainless steel by using nano MoS_2_ reinforced vegetable cutting fluid. Procedia Soc. Behav. Sci..

[10] Kamata Y., Obikawa T. (2007). High speed MQL finish-turning of inconel 718 with different coated tools. J. Mater. Proc. Technol..

[11] Deiab I., Raza S.W., Pervaiz S. (2014). Analysis of lubrication strategies for sustainable machining during turning of Titanium Ti-6Al-4V alloy. Procedia CIRP.

[12] Busch K., Hochmuth C., Pause B., Stoll A., Wertheim R. (2016). Investigation of cooling and lubrication strategies for machining high-temperature alloys. Procedia CIRP.

[13] Vagnorius Z., Sørby K. (2011). Effect of high-pressure cooling on life of SiAlON tools in machining of Inconel 718. Int. J. Adv. Manuf. Technol..

[14] Bruschi S., Bertolini A., Medea F., Ghiotti A. (2016). Influence of the machining parameters and cooling strategies on the wear behavior of wrought and additive manufactured Ti6Al4V for biomedical applications. Tribol. Int..

[15] Pereira O., Rodríguez A., Fernández-Abia A.I., Barreiro J., de Lacalle L.N.L. (2016). Cryogenic and minimum quantity lubrication for an eco-efficiency turning of AISI 304. J. Clean. Prod..

[16] Fernandez-Valdivielso A., de Lacalle L.N.L., Urbikain G., Rodriguez A. Detecting the key geometrical features and grades of carbide inserts for the turning of nickel-based alloys concerning surface integrity. Proc. Inst. Mech. Eng. Part C J. Mech. Eng. Sci..

